# Draft Genome Sequence of Anaerobic Fermentative Bacterium *Anaeromicrobium sediminis* DY2726D

**DOI:** 10.1128/genomeA.00002-18

**Published:** 2018-02-08

**Authors:** Jing Zhang, Junxi Hu, Xiaobo Zhang, Xiang Zeng

**Affiliations:** a Key Laboratory of Marine Biogenetic Resources, the Third Institute of Oceanography SOA, Xiamen, Fujian, People's Republic of China; b Xinjiang Medical University, Urumqi, Xinjiang, People's Republic of China; c Minnan Normal University, Zhangzhou, Fujian, People's Republic of China

## Abstract

Here, we report the draft genome sequence of *Anaeromicrobium sediminis* DY2726D, isolated from a west Pacific Ocean sediment sample. The genome comprises 4,710,590 bp in 56 contigs, with a G+C content of 31.2%. A total of 3,811 protein-coding sequences were predicted. The genome annotation revealed that DY2726D may represent a marine type of *Clostridiaceae*.

## GENOME ANNOUNCEMENT

The *Clostridiaceae* family includes a large proportion of anaerobic fermentative microorganisms, and some species have been frequently isolated from marine environments, including *Clostridium bryantii* (1), *Caloranaerobacter azorensis* MV1087^T^ (2), and *Wukongibacter baidiensis* DY30321^T^ (3). *Anaeromicrobium sediminis* DY2726D was isolated in 2013 from a deep-sea sediment sample collected in the west Pacific Ocean. *Anaeromicrobium sediminis* DY2726D was found to be a member of the family *Clostridiaceae* and to be most closely related to species of the genera *Clostridium* and *Alkaliphilus*. The most closely related strain is *Alkaliphilus transvaalensis* SAGM1 (4), with which *A. sediminis* DY2726D has 90.0% 16S rRNA gene sequence similarity. DY2726D is the type strain of the new genus *Anaeromicrobium* (5). Here, we report the draft genome sequence of *A. sediminis* DY2726D, the first released *Anaeromicrobium* genome sequence.

The DY2726D genome was sequenced by using the Illumina HiSeq 2000 platform (Majorbio Co., Ltd., Shanghai, China). Paired-end reads with an average length of 500 bp and total read size of ~300 Mbp were assembled by using SOAPdenovo version 2.04 (6), and gap filling between contigs and the remaining gaps between scaffolds were closed by using GapCloser version 1.12 (7).

The assembled draft genome contained 4,710,590 bp in 56 contigs with an average of 20× coverage and 31.2% G+C content. The open reading frames (ORFs) were analyzed using Glimmer 3.02 (8), and all predicted ORFs were then searched by BLAST against all proteins from complete microbial genomes by using the NCBI Prokaryotic Genome Annotation Pipeline (9). tRNAscan-SE (version 1.3.1) was used to identify the tRNA genes (10), and rRNA identification was performed by Barrnap 0.4.2 (11). Classifications of some predicted genes and pathways were analyzed by using the COG database (12) and the KEGG database (13, 14). A total of 4,258 putative genes, of which 3,811 were protein-coding genes, and a total of 5 rRNAs (four 5S rRNA genes and one 16S rRNA gene) and 45 tRNAs were found in the genome. The 45 tRNAs may be involved in the transfer of 19 amino acids.

The draft genome sequence of DY2726D included a normal complement of genes for metabolic enzymes involved in glycolysis, carbohydrate utilization, and biosynthesis of amino acids and fatty acids, as well as essential genes for nucleotide metabolism, transcription, and replication. *A. sediminis*, which is a heterotrophic anaerobic bacterium, harbors genes that encode a large number of hydrolases, such as glucosidase, β-galactosidase, chitinase, cellulose, protease, peptidase, and related substrate transporters. It is also able to grow heterotrophically on glucose, fructose, fumarate, or malate but may also use glycine or betaine as energy and carbon sources, as gene clusters coding for a glycine and betaine reductase are encoded. *A. sediminis* possesses a complete tricarboxylic acid (TCA) cycle, a modified Embden-Meyerhof pathway to glycolysis, and metabolism pathways for proteins and carbohydrates. Genome analysis also revealed the presence of genes encoding the Rnf complex and genes involved in quinone biosynthesis but the absence of cytochrome *c* synthesis genes. The respiration system is represented by membrane-bound iron-only hydrogenase and F1F0-type ATP synthase.

The genome of strain DY2726D will contribute to the increasing scope and depth of the *Clostridiaceae* genome database and may shed light on the marine environmental adaptation mechanisms and evolutionary history of *Clostridiaceae*.

### Accession number(s).

This whole-genome shotgun project has been deposited in DDBJ/ENA/GenBank under the accession no. NIBG00000000. The version described in this paper is the first version, NIBG01000000. Data also have been deposited under BioProject no. PRJNA387640 and RefSeq accession no. NZ_NIBG00000000.
